# Trichomes in Plant Defence, Development and Metabolic Integration Under Environmental Stress

**DOI:** 10.1111/pce.70752

**Published:** 2026-07-16

**Authors:** Debdatta Chatterjee, Madhabi Das, Sanjib Patra, Srijib Chatterjee, Amit Roy, Arunava Mandal

**Affiliations:** ^1^ Department of Genetics University of Calcutta Kolkata India; ^2^ Faculty of Forestry and Wood Sciences Czech University of Life Sciences Prague Czech Republic

**Keywords:** bioeconomy, biofactories, biotechnological innovation, biotic stress, plant protection, plant trichomes, secondary metabolites, sustainable agriculture

## Abstract

Among various plant defence strategies, trichomes are tiny hair‐like structures on the epidermis that aid in defence, environmental adaptation and the production of valuable secondary metabolites. This review features trichomes as frontline guards for plant survival, integrating their structural, physiological and metabolic roles in safeguarding against environmental adversity. We explore the developmental biology of trichomes, emphasising a multilayered regulatory network governed by transcriptional hierarchies, hormonal crosstalk, epigenetic remodelling and post‐transcriptional modulation. Trichomes, divided into glandular and non‐glandular types, function as physical shields and dynamic chemical interfaces, mediating defence against herbivores, pathogens, UV radiation and abiotic stresses such as drought and heavy metal exposure. Notably, glandular trichomes serve as specialised biosynthetic centres, producing high‐value secondary metabolites, such as terpenoids, alkaloids and phenolics, which are increasingly harnessed in sustainable agriculture and green biotechnologies. Considering the broader context of climate resilience and bioeconomic innovation, this review describes trichomes as multifunctional and evolvable units of plant adaptation. It also highlights emerging research avenues such as single‐cell omics, chromatin dynamics, metabolic and genetic engineering, which offer promising strategies for exploiting trichomes as a foundation for next‐generation crop improvement and sustainable metabolic engineering.

## Summary statement

This review provides a comprehensive synthesis of the structural, developmental, defensive and metabolic roles of plant trichomes under both biotic and abiotic stress. Our manuscript integrates current knowledge on transcriptional regulation, phytohormone signalling, epigenetic control, regulatory RNAs and secondary metabolite biosynthesis, while highlighting the importance of glandular and non‐glandular trichomes as multifunctional interfaces between plants and their environments. By linking trichome biology with climate resilience, sustainable agriculture, crop improvement and metabolic engineering, we present a timely and valuable perspective on the potential of trichomes as targets for next‐generation plant biotechnology.

## 1 Introduction

Plants have evolved diverse structural and biochemical strategies to endure and flourish under a wide range of biotic and abiotic stresses, including herbivory, insect attack, elevated temperatures, UV exposure, drought and high salinity (Figure 1). One of these adaptive features is trichomes, tiny hair‐like outgrowths that develop primarily from epidermal cells and can be found on nearly all aerial parts of plants, including leaves, stems and floral organs. Leaf and stem trichomes are mainly associated with plant defence, whereas flower trichomes are associated with pollination biology (Zheng et al. 2025). Although trichomes are not essential for viability under optimal growth conditions, they play a range of vital protective roles against biotic and abiotic stresses. Moreover, the combined effects of biotic and abiotic stress can result in a distinct stress response by altering trichome‐related gene expression (Mittler 2006). For example, trichome density in *Helianthus annuus* increases by 79% under cadmium stress and by 53.5% under herbivory, but combined stress decreases trichome density without affecting deterrence against insect pests (Grossman et al. 2024). Therefore, choosing the right candidate gene using the stress matrix is essential to address the crosstalk‐associated trade‐off for enhanced productivity. Trichomes represent the primary line of defence in plants, protecting them through physical and chemical means against environmental challenges (Harada et al. 2010). Trichomes can be categorised based on their cellular composition (unicellular or multicellular), morphology (straight with little branching), spiral (corkscrew‐shaped or hooked with a curved apex), shape (tortuous, simple, peltate or stellate) and secretory ability (glandular or non‐glandular) (X. Wang et al. 2021). A single plant species may possess multiple types of trichomes; for instance, tomato plants have seven distinct trichome types (I–VII), with types I, IV, VI and VII classified as glandular and types II, III and V as non‐glandular (Watts and Kariyat 2021; Mcdowell et al. 2010). The structure of glandular trichomes varies; types I and IV have single‐celled heads, whereas types VI and VII contain four and eight‐head cells, respectively (Huchelmann et al. 2017). This variation in head cell number clearly shows its functional significance, as multicellular heads provide greater storage capacity for secondary metabolites and biosynthetic output (Feng et al. 2021). The diversity of trichomes across plant species reflects their varied functions in different environments. Non‐glandular trichomes help plants cope with heat stress, increase their resistance to cold temperatures, promote seed dispersal, aid in water absorption and shield plant tissues from biological threats, including insect herbivores and UV radiation (Huchelmann et al. 2017; Karabourniotis et al. 1993). Conversely, glandular trichomes provide chemical protection against herbivores and pathogens by secreting enzymes that can attract animals through the release of secondary metabolites (Glas et al. 2012). Numerous metabolites and compounds secreted by glandular trichomes have significant value in the pharmacological and biochemical industries (Wagner 1991; T. Chen, Ma, et al. 2025). The release of metabolic compounds results either via diffusion or mechanical stimulation. In the peppermint plant, for example, menthol can diffuse through the cuticle of glandular trichomes. This diffusion can be influenced by external factors, such as temperature, where increasing the temperature from 22°C to 39°C significantly increased the amount of menthol from 2% to 12% of the total product of study (Shotipruk et al. 2001). Additionally, rupture of the cuticle due to insect locomotory sensation or other environmental stimuli results in the release of compounds from the extracellular storage cavity between the cuticle and cell wall (Popowski et al. 2025). In plants such as *Solanum lycopersicum* (tomato), *Nicotiana tabacum* (tobacco) and *Cannabis sativa*, glandular trichomes produce secondary metabolites, including terpenoids, flavonoids and alkaloids, which hold economic significance (Lange and Turner 2013). These compounds serve dual functions: they provide defence mechanisms for the plant and are utilised in various applications, including natural pesticides, flavourings, fragrances and therapeutic agents. The identification and detailed characterisation of the genes that initiate, regulate and drive the growth of these glandular structures is therefore essential. The formation and development of trichomes are tightly coordinated by intricate molecular mechanisms that dictate cell‐fate specification and morphogenesis (C. Yang and Ye 2013). Its development begins with the selection of a single epidermal cell to adopt the trichome fate through initiation, endoreplication and branching followed by maturation. This decision is regulated by the GLABRA1 (GL1), GLABRA3 (GL3) and TRANSPARENT TESTA GLABRA1 (TTG1) complex, which activates the transcriptional programme required for trichome initiation and is precisely regulated by mobile inhibitors such as CAPRICE (CPC) and TRIPTYCHON (TRY) to assure appropriate spatial patterning (Zhao et al. 2008). GLABROUS INFLOROSCENCE STEM (GIS) is activated by GA repressor protein SPY (SPINDLY) and DELLA repressor GIBBERELLIC ACID INSENSITIVE (GAI) which further induces with trichome initiation complex. Several ZINC FINGER PROTEINs (ZFP) such as ZFP5 and ZFP8 interact with GL3 and promote trichome initiation. On the other hand, TRICHOME RELAYED PROTEIN (TRP) are group of ZFPs which hinders the process (Kim et al. 2018). Now, the trichome differentiation is governed by TTG proteins which interacts with GL3 and boost trichome differentiation. Here, TTG2, a WRKY transcription factor, binds particularly to the minimal promoter of TRY gene and also interacts with GL1/GL2/TTG1 for enhanced expression of trichome patterning genes (Pesch et al. 2014). However, this process is negatively regulated by CPC and TRY which bind to the GL3/EGL3 complex and renders them inactive (Wada et al. 1997). Then, through the coordinated action of genes like STICHEL (STI), ZWICHEL(ZWI) and SPIKE1 (SPK1) microtubule assembly takes place which regulate cell expansion and branching, the growing trichome elongates and adopts its distinctive form, which is frequently branched or unbranched. Although a MYB transcription factor NOEK (NOK) negatively regulates ANGUSTIFOLIA (AN) and hinders the process of trichome branching (Ilgenfritz et al. 2003; Han et al. 2022; Jakoby et al. 2008). Phytohormones such as gibberellins (GA), cytokinins (CK) and jasmonates (JA) act as key regulators by enhancing the expression of trichome‐promoting genes (J. Li et al. 2021). This process involves a complex interplay of transcription factors, hormonal signals, epigenetic modifications and environmental stimuli. A recent transcriptome analysis also revealed that in *N. tabacum*, a number of transcription factor governs the gene expression involved in glandular trichome formation and can be a new lead in the biotechnology for enhanced secondary metabolite production (Nautiyal et al. 2020). Although numerous studies have described trichomes as physical barriers and secretory structures involved in stress mitigation, their functional diversity across development, defence, abiotic‐stress adaptation and specialised metabolism remains fragmented across species and trichome types. A key knowledge gap is the lack of an integrated framework explaining how molecular regulation, environmental cues and metabolic outputs jointly shape the functional diversity of glandular and non‐glandular trichomes. This review addresses this gap by synthesising current knowledge on trichome development, defensive roles and secretory activities, while highlighting future opportunities for trichome‐based crop improvement, biotechnology and sustainable agriculture.

**Figure 1:**
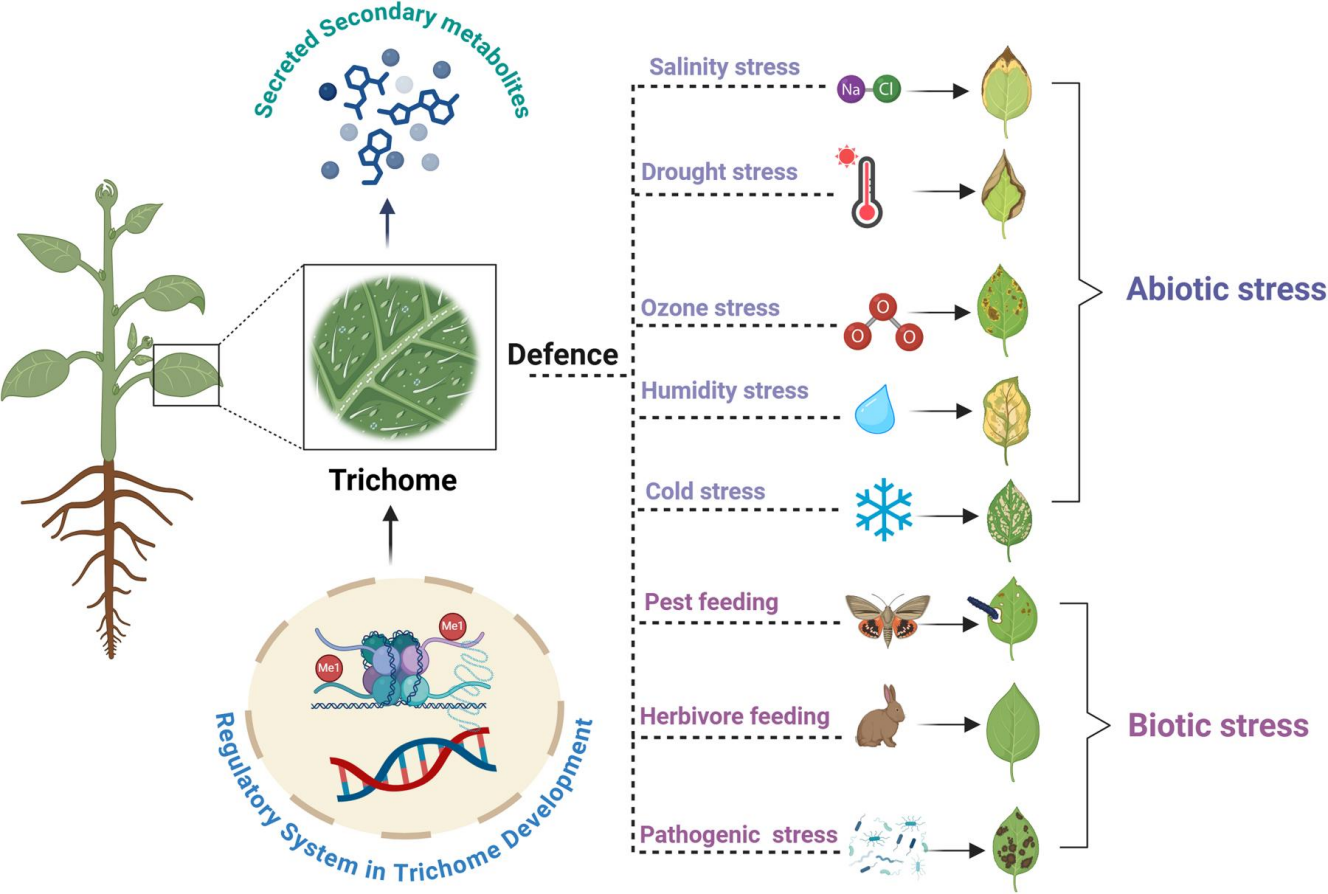
Trichome‐mediated plant defence responses to abiotic and biotic stresses. Diagrammatic representation of trichome‐mediated defence strategy under various abiotic and biotic stress conditions. Plants, after experiencing abiotic stresses such as drought, cold, salt and heat, as well as biotic stresses such as insect pests and herbivores, activate the regulatory system involved in trichome development. Subsequently, they either produce secondary metabolites or increase trichome density to overcome the adverse situation (Nawaz et al. 2023; Hussain et al. 2023). Created in Biorender.com.

## 2 Molecular Underpinnings of Trichome Development

### 2.1 Core Transcriptional Regulators of Trichome Initiation

*Arabidopsis thaliana*, an extensively utilised model organism in plant biology, has been well studied for its development of unicellular, non‐glandular trichomes. This process is tightly regulated by a network of transcription factors, in which some act as positive regulators to promote trichome formation, while others act as negative regulators to inhibit it (Szymanski et al. 2000) (Figure 2 and Table 1). MYB proteins (GL1, MYB23, MYB5), basic helix‐loop‐helix (bHLH) proteins (GL3, EGL3) and the WD40‐repeat protein TTG1 are the essential transcription factors that make up the MYB–bHLH–WD40 (MBW) complex, which is at the centre of this pathway (Zhao et al. 2008; Mengxuan et al. 2025). This complex triggers downstream targets, such as GLABRA2 (GL2), a homeodomain‐leucine zipper (HD‐ZIP IV) transcription factor, which is necessary for trichome initiation, morphogenesis and branching (S. Wang and Chen 2008). The loss of GL2 results in reduced trichome numbers and branching defects (Di Cristina et al. 1996). A recent report suggests that *SlHDZIV3* induces long trichome formation only on tomato leaves, while SlHDZIV9 affects a broader tissue range, including flowers (M. Li et al. 2025). The bHLH proteins GL3 and EGL3 also regulate endoreduplication and determine cell size; together with GL1 and TTG1, they form a central transcriptional network crucial for proper trichome development (Payne et al. 2000). GL1 was one of the first identified regulators; its knockout leads to glabrous leaves, whereas overexpression unexpectedly suppresses trichome identity (Bloomer et al. 2012). A well‐known mathematical model, the activator‐depletion (AD) and activator–inhibitor (AI) models, helps understand the molecular meshwork underlying trichome patterning (Balkunde et al. 2020). According to this model, AI promotes the expression of repressors, which then repress nearby activators and hamper trichome initiation. Whereas AD establishes trichome patterning by repressing the activators. For example, TTG1 is part of the activator complex; it moves between cells in a non‐cell‐autonomous manner, preferentially migrating to cells rich in GL3. This movement forms the basis of the activator‐depletion model, in which the local enrichment of TTG1 activates trichome development in select cells, while its depletion in neighbouring cells renders them less competent, ensuring proper trichome spacing. Alternatively, the activator–inhibitor model suggests that activators trigger the production of mobile inhibitors, which then move to neighbouring cells and block trichome formation. The network's inhibitory arm includes R3‐MYB transcription factors such as TRY, CPC and ENHANCER TRY AND CPC1 (ETC1) (Kirik et al. 2004). These proteins compete with GL1 for binding to EGL3 and GL3, breaking the activator complex and preventing neighbouring cells from initiating trichomes (Morohashi et al. 2007). In *Arabidopsis*, APETALA 2‐like transcription factor TARGET OF EAT1 (TOE1) and TOE2 also promote trichome development on the inflorescence stem by interacting with GIS‐family proteins (C2H2 zinc finger domain protein) and thereby regulating GL1/GL3 expression. Using mutant analysis, overexpression lines and protein interaction assays (Y2H, BiFC), the researchers confirmed their role downstream of gibberellin signalling (Y. Liu et al. 2023). Like *Arabidopsis* trichomes, cotton trichomes (fibres) are non‐glandular and single‐celled. However, it entails a more intricate regulatory network consisting of four overlapping stages (Y. Wang et al. 2022). It contains transcription factors such as MYB (e.g., *GhMYB109*, *GhMYB25*), bHLH (*GhMYC1*), HOX (*GhHD1*, *GhHOX3*) and NAC (*GhFSN1*), as well as hormonal signals, including reactive oxygen species (ROS) and ethylene. Positive regulators promote fibre initiation and elongation, while negative regulators, such as *GhJAZ2* and *GhCPC*, inhibit the process (Z. Wang et al. 2019). Trichomes in the tomato (*S. lycopersicum*) are multicellular and can be either glandular or non‐glandular. While some regulatory elements, such as *SlGL3* and *SlTRY*, are conserved in *Arabidopsis*, tomato‐specific factors, including Woolly (Wo) and Hair (H), play key roles in trichome initiation and type determination. Jasmonate signalling interacts with transcription factors, such as *SlMYC1*, to regulate the development of glandular trichomes (M. Wu et al. 2023). Researchers discovered that the *SlMixta*‐like gene functions as a negative regulator of trichome formation in domesticated tomatoes. By developing introgression lines after repeated cross over between domesticated tomato (M82) and the wild species *Solanum pennellii* (LA716) it was noticed that *SlMixta*‐like gene(a MYB type transcription factor) acts as a repressor for domasticated tomatoes and its higher expression level due to the presence of a short motif sequence in the 5′ UTR of M82 allele reduces trichome number which is absent in the LA716 allele of the wild species of tomato (Andreuzza 2020). Studies in *Artemisia annua* by Xie et al. (2021) have identified *AaGSW2*, a WRKY transcription factor regulated by homeodomain proteins *AaHD1* and *AaHD8*, as an essential regulator of glandular‐secreting trichomes (GSTs), which are critical for the production of the antimalarial compound artemisinin. Additionally, further studies demonstrate that *AaGSW2* homologues from mint can also increase GST formation and artemisinin content when expressed in *A. annua*, indicating a mechanism that is retained in the control of peltate trichomes across plant families (Asteraceae and Lamiaceae) (Xie et al. 2021). In *Arabis alpina*, a forward EMS (ethylmethanesulfonate) mutagenesis screen revealed conserved regulatory components but also notable divergence. While *AaTTG1 and AaGL3* were critical for trichome formation, *AaGL1* mutants were not recovered, and its overexpression reduced trichome density—opposite to what is observed in *A. thaliana*. Moreover, *A. alpina* also lacks accessory cells and has two distinct trichome size classes. TRY function remains conserved, and GL2 appears to regulate only small trichomes, suggesting size‐specific genetic regulation (Chopra et al. 2019). A detailed transcriptomic study revealed that glandular trichome in *N. tabacum* expressed some key genes and transcription factors such as CBTS (cembratrien‐diol synthase), CYP71D16 (cytochrome P450), CPS2 (copalyl diphosphate synthase), ABS (abienol synthase), oxidosqualene cyclases and beta‐amyrin oxidase‐like genes, along with transcription factors from the MYB, HD‐ZIP, ERF, WRKY, TCP, SRS and DOF families, that significantly enhance the secondary metabolic pathway associated genes (Nautiyal et al. 2020). Very recently, a genome‐wide study in cucumber revealed that B3 family transcription factor is associated with maintaining glandular trichome structure.

**Figure 2:**
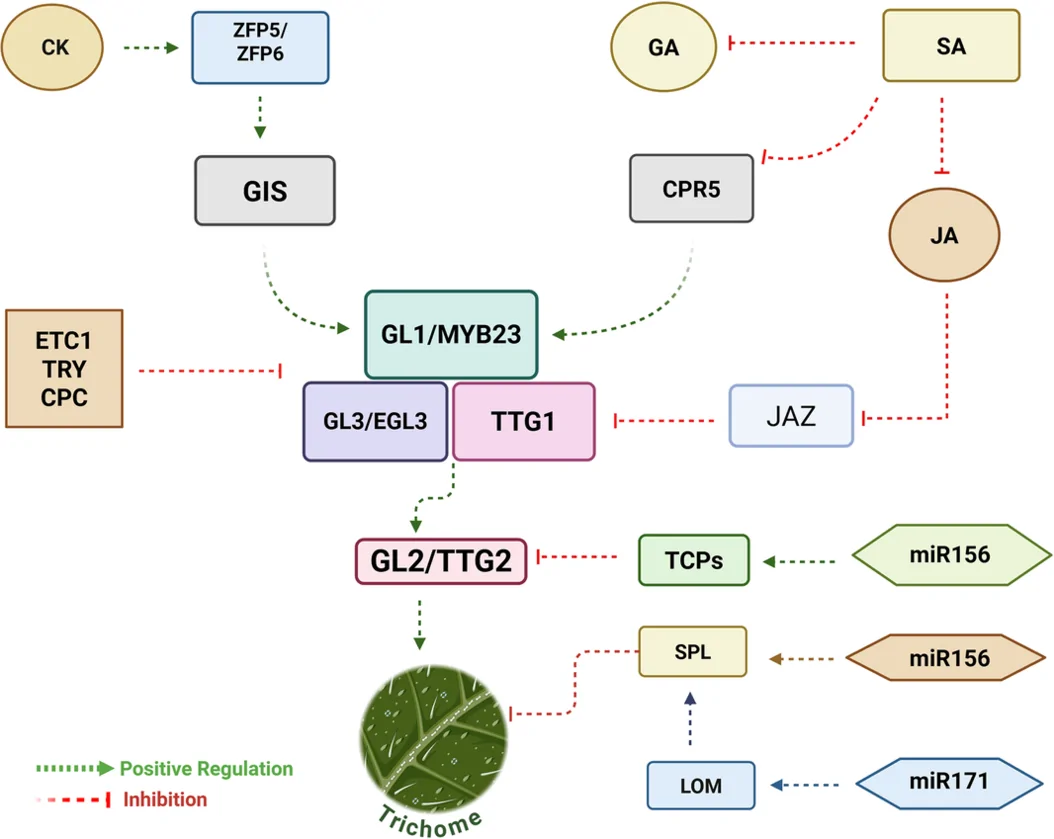
Regulatory network of hormonal, transcriptional and microRNA‐mediated control of trichome development. Gibberellic acid (GA), salicylic acid (SA) and jasmonic acid (JA) interact through a complex hormonal crosstalk involving CPR5 and JAZ proteins, which induce trichome formation. Cytokinin (CK) promotes trichome initiation through ZFP5/ZFP6 and GIS, which activate the core transcription factor GL1/MYB23. The MYB–bHLH–WD40 (MBW) complex, comprising GL1/MYB23, GL3/EGL3 and TTG1, induces the expression of downstream regulators GL2 and TTG2, thereby promoting trichome formation. Negative regulators, including ETC1, TRY and CPC, suppress MBW complex activity and inhibit trichome development. MicroRNA‐mediated regulation also contributes to trichome development: miR156 targets TCP and SPL transcription factors, while miR171 regulates LOM, which, in turn, influences SPL activity. Green dashed arrows indicate positive regulation, whereas red dashed lines indicate inhibition. The diagram summarises key genetic and hormonal pathways controlling trichome initiation and differentiation (Han et al. 2022). gl1, glabra1; gl2, glabra2; gl3, glabra3; ttg1, transparent testa glabra1; cpc, caprice; try, triptychon; sti, stichel; zfp5/6/8, zinc finger protein; cpr5, constitutive expressor of pathogenesis related genes 5; gis2, glabrous inflorescence stems; etc1, enhancer of try and cpc1 gene; jaz, jasmonate zim‐domain proteins; tcp, teosinte branched1/cycloidea/pcf; spl, squamosa promoter binding 245 protein‐like; lom, lost meristems. Created in Biorender.com. [Color figure can be viewed at wileyonlinelibrary.com]

**Table 1:** Transcriptional regulators of trichome formation in various plant species.

Plant species	Trichome type	Key transcription factors	Notes	References (DOI)	References (author name)
*Arabidopsis thaliana*	Unicellular, non‐glandular	MYB group (GL1, MYB23, MYB5); bHLH group (GL3, EGL3); WD40 (TTG1); HD‐ZIP IV (GL2); R3‐MYBs (TRY, CPC, ETC1); TOE1, TOE2, GIS‐family	MBW complex is central; GL2 for morphogenesis; TRY/CPC inhibit; TTG1 is mobile, contributes to spacing	doi: 10.1016/j.plantsci.2025.112385, doi: 10.1242/dev.016873	Mengxuan et al. (2025), Zhao et al. (2008)
*Gossypium hirsutum*	Unicellular, elongated	MYB family (GhMYB109, GhMYB25); bHLH (GhMYC1); HOX (GhHD1, GhHOX3); NAC (GhFSN1); repressors (GhJAZ2, GhCPC)	Hormonal crosstalk with ROS and ethylene; both positive and negative regulators present	doi: 10.1111/pbi.13167	Z. Wang et al. (2019)
*Solanum lycopersicum*	Multicellular, glandular and non‐glandular	Conserved regulators (SlTRY, SlGL3); unique regulators (Wo ‐ HD‐ZIP, H ‐ zinc finger); MYB (SlMixta‐like); bHLH (SlMYC1)	Jasmonate signalling and tomato‐specific genes regulate trichome patterning	10.1016/j.devcel.2023.01.009	M. Wu et al. (2023)
*Artemisia annua*	Glandular‐secreting trichomes (GSTs)	AaGSW2 (WRKY); AaHD1, AaHD8 (homeodomain); mint homologues of AaGSW2	Regulates artemisinin production; regulatory conservation with Mentha in peltate trichomes	doi: 10.1093/jxb/eraa523	Xie et al. (2021)
*Arabis alpina*	Unicellular, lacking support cells	Core components (AaTTG1, AaGL3); TRY (conserved); GL2 (restricted to small trichomes); GL1 (limited or opposite role)	Two trichome size classes; GL1 function and trichome accessory structure, differ from those of *Arabidopsis*	doi: 10.1073/pnas.1819440116	Chopra et al. (2019)

### 2.2 Phytohormone Signalling in Trichome Patterning

Trichome development involves genetic regulators and a dynamic network of phytohormone signalling. These include gibberellins (GA), cytokinins (CK), jasmonic acid (JA), salicylic acid (SA), auxin, ethylene and brassinosteroids (BRs) and abscisic acid (ABA) (J. Li et al. 2021) (Figure 2 and Table 2). These hormonal signals regulate cell fate determination, spatial arrangement and morphological differentiation of trichomes, primarily in *A. thaliana*, where trichomes act as a model system because of their distinct development pattern and visibility (Gan et al. 2007). Gibberellins are essential for the development of *Arabidopsis* and many other species, as they initiate and promote the growth of branch trichomes. Studies have shown that GAs promote trichome formation in *Arabidopsis* by upregulating GL1, a crucial MYB transcription factor that defines trichome cell fate. They discovered a correlation between GA levels and trichome quantity and branching using GA‐deficient (ga1‐3), GA‐response (spy‐5) mutants and uniconazole therapy. RT‐PCR and GUS reporter tests verified that GAs activate GL1 transcriptionally (Perazza et al. 1998). CKs work with GAs to positively regulate trichome formation, especially in inflorescence stems where trichome numbers surge during growth. Cytokinin‐inducible transcription factors, such as GIS2 and ZFP8, play a crucial role in promoting trichome formation, acting upstream of GL1 and closely linked to the GA‐GIS pathway. When cytokinin signalling or components, such as SPINDLY (SPY) (which also responds to GA), are disrupted, this coordination is weakened, leading to reduced trichome development (L. Sun et al. 2015; An et al. 2011; Greenboim‐Wainberg et al. 2005). Jasmonic acid serves as a widespread and positive regulator of trichome initiation across various plant species, including *Arabidopsis*, *A. annua*, cucumber, tomato and cotton. JA signalling enhances the activity of the TTG1–GL1–GL3/EGL3 complex, promoting endoreduplication in trichome progenitor cells, which is crucial for trichome size and branching. Mutants deficient in JA exhibit reduced trichome density, whereas JA treatment or wounding can enhance trichome production (Yoshida et al. 2009). Jasmonate ZIM‐domain (JAZ) proteins, important repressors in this pathway, bind to GL1 and GL3/EGL3 to prevent trichome initiation. When JA builds up, JAZ proteins are broken down, which permits the action of transcription factors that promote trichomes (HU et al. 2016). For example, in cucumber, the *CsLOX* gene, a glandular trichome‐specific gene, positively regulates the jasmonic acid pathway and boosts trichome density (S. Yang, Feng, et al. 2025). Additionally, JA signalling is fine‐tuned by interactions with GA and CK pathways to adjust trichome development. Salicylic acid suppresses trichome development by decreasing trichome density and reducing the number of trichomes on newly formed leaves (Fambrini and Pugliesi 2019). SA not only directly diminishes trichome production but can also counteract the positive effects of JA and GA, highlighting a strong negative crosstalk and a balancing mechanism in the plant's response to environmental and developmental signals. Gain‐of‐function mutants or transgenic lines exhibiting elevated SA levels further substantiate the suppressive role of this hormone on trichome patterning (Traw and Bergelson 2003). Ethylene regulates trichome development by influencing endoreduplication, cell division polarity and microtubule dynamics, affecting trichome branching, shape and abundance. Ethylene signalling mutants, such as etr2‐3 in *Arabidopsis*, exhibit defects in trichome morphology, emphasising the hormone's role in cytoskeletal organisation (Plett et al. 2009). Ethylene‐responsive transcription factors integrate signals from stress and hormone pathways, facilitating adaptive changes in trichome form and distribution. For example, ethylene‐activated very long chain saturated fatty acid (VLCFAs) significantly increases cotton fibres, whereas VLCFAs lose their potential in the presence of ethylene biosynthesis inhibitors, resulting in reduced fibre length (Y. M. Qin et al. 2007). In cotton fibre growth, brassinosteroids are essential, especially for fibre elongation. Exogenous BR application promotes fibre elongation, while inhibitors of BR biosynthesis suppress this trait, demonstrating the hormone's direct impact on trichome growth fibre elongation and BR biosynthesis are improved by overexpression of the bHLH transcription factor *GhFP1* (Z. H. Liu et al. 2020). Fibre development and BR signalling are facilitated by 14‐3‐3 proteins such as Gh14‐3‐3L. Silencing 14‐3‐3 proteins leads to shorter fibres, but treatment with 2,4‐epibrassinolide can restore normal fibre length (Z. Zhang et al. 2010). Moreover, the regulation of trichome formation at the transcriptional and metabolic levels is improved by the regulatory interaction of BRs with other hormones, including auxin and ethylene. Auxin primarily influences trichome initiation and elongation by regulating the expression and activity of auxin response factors (ARFs) and transporters (Yamauchi et al. 2019; Yuan et al. 2021). As shown in tomato and cotton, auxin accumulation increases trichome initiation, whereas auxin transport blockage decreases initiation and elongation. Auxin regulates trichome development through ARF proteins and Aux/IAA repressors. Manipulation of these proteins, such as inhibiting AUX/IAA proteins or knocking down ARFs, leads to measurable changes in trichome number and type, often in coordination with other hormones, like gibberellins (GA) (Deng et al. 2012). Strigolactones (SLs) are carotenoid‐derived plant hormones which crosstalk with gibberellins, auxin and cytokinin and help in trichome development (F. Wu et al. 2022). For instance, exogenous application of strigolactone increases the fibre length in *Gossypium barbadense* (L. Chen, Xu, et al. 2025; Wen et al. 2024). It was noticed that strigolactones, after being positively triggered by gibberellin, upregulate four different transcription factors *GhBLH51*, *GhB9SHZ1*, *GhNAC100‐2* and *GhGT2* which target cellulose and fatty acid synthesis genes and thereby help in good quality cotton fibre (Tian et al. 2022).

**Table 2:** Hormones involved in trichome development.

Hormone	Key transcription factors/targets	Functional role	Reference (DOI)	Reference (author name)
Gibberellin	GL1, GIS, ZFP5, ZFP8	Initiation and branching of trichome	10.1104/pp.117.2.375	Perazza et al. (1998)
Cytokinin	GIS2, ZFP8, GIS	Work along with GA and control initiation	10.4161/psb.6.12.18120, 10.1105/tpc.104.028472, 10.1111/nph.13218	An et al. (2011), Greenboim‐Wainberg et al. (2005), L. Sun et al. (2015)
Jasmonic acid	JAZ, GL3, GL1, TTG1, MYB25‐like	Promotion of trichome density, endoreduplication	10.1242/dev.030585, 10.1111/tpj.13273	Yoshida et al. (2009), Hu et al. (2016)
Salicylic acid	—	Suppression of initiation and density	10.3390/plants8080253	Fambrini and Pugliesi (2019)
Ethylene	ETR2, microtubule‐related genes	Branching, morphogenesis	10.1093/jxb/erp228	Plett et al. (2009)
Brassinosteroid	GhFP1, CPD, DWF4, DET2	Fibre/trichome elongation	10.1111/nph.16301, 10.1093/jxb/erq155	Z. H. Liu et al. (2020), Z. Zhang et al. (2010)
Auxin	ARF3, AUX/IAA, PIN, TCP14	Initiation, elongation, fine‐tuning	doi: 10.1111/pbi.13448	Yamauchi et al. (2019), Yuan et al. (2021)
Strigolactones	GhGT2, GhBLH51, GhB9SHZ1, GhNAC100‐2	Activate genes associated with very long chain fatty acid and cellulose, and develop good quality cotton	doi: 10.1093/plcell/koac270	Tian et al. (2022)

### 2.3 Epigenetic Regulation Underlying Trichome Development

Epigenetic modifications are crucial for plant trichome development, as they regulate gene expression without altering the underlying DNA sequence (Y. Wang, Zhou, et al. 2022). Histone modifications that directly affect chromatin structure and transcriptional results include acetylation (ac), methylation (me), ubiquitination (ub) and phosphorylation. In *Arabidopsis*, the histone acetyltransferase GCN5, a component of the SAGA complex, may promote trichome branching by modulating TRY, a key negative regulator of trichome patterning. Furthermore, *AtGCN5* is involved in both the early and late phases of trichome formation by controlling histone acetylation at the promoters of GL1, GL2, GL3 and CPC, genes essential for trichome initiation (Y. Dong et al. 2023). Histone deacetylases (HDACs), on the other hand, remove these acetyl groups, leading to transcriptional suppression and tighter chromatin packing. In cotton, *GhHDA5* serves as a key HDAC during fibre initiation. Its knockdown results in impaired fibre formation, confirming the conservation of histone acetylation dynamics in epidermal differentiation across species (Kumar et al. 2018). Histone methylation is also crucial for trichome development. In *Arabidopsis*, JMJ29 demethylase targets H3K9me1/2, which has been shown to demethylate the GL3 locus, thereby promoting its expression and initiating trichome formation (Hung et al. 2020). Another chromatin‐associated element that controls fibre initiation and early elongation in cotton is the histone variant *GhH2A12*, which regulates genes associated with the cell cycle. Overexpression of *GhH2A12* delays fibre development and produces shorter fibres, linking chromatin structure to cell proliferation and expansion dynamics (Hao et al. 2014). DNA methylation at CG, CHG and CHH sites is a key epigenetic mark influencing chromatin conformation and transcriptional activity. During cotton fibre (a form of trichome) development, dynamic changes in CHH methylation and reductions in CG methylation in intergenic and regulatory regions are observed, affecting gene expression patterns necessary for proper differentiation. In tomatoes, the SlIDM3‐HAP chromatin remodelling complex orchestrates sequence‐context‐dependent methylation changes; loss‐of‐function mutants in these components exhibit altered trichome numbers and types due to shifted methylation landscapes (Nyikó et al. 2025). In *C. sativa*, epigenetic study revealed that promoters of terpenoids and cannabinoid genes, defence response genes and some metabolic transporter genes present in the trichome possess H3K4me3 and H3K56ac marks, which results in active transcription (Conneely et al. 2024). RNA methylation is another essential post‐transcriptional modification that begins to regulate plant development and stress response (Shinde et al. 2023). *N* ^6^ methyladenosine (m^6^A) is a common modification recognised by some m^6^A reader proteins and is involved in plant development. For example, YTH domain containing m^6^A reader protein ECT2 in *Arabidopsis* binds preferentially to UGUA motif of 3′UTR of specific transcripts, which are associated with correct trichome branching. In contrast, mutated ECT2 results in transcript degradation and abnormal trichome patterning (Wei et al. 2018).

### 2.4 Regulatory RNAs in Trichome Development

Recent research has demonstrated that small regulatory RNAs, specifically microRNAs (miRNAs) and small interfering RNAs (siRNAs), play a pivotal role in regulating the activity of key genes following transcription, thereby influencing trichome formation, patterning and morphogenesis (C. Chen et al. 2018) (Figure 2). miRNAs are typically about 21 nucleotides in length and function by binding to complementary mRNA sequences, leading to mRNA cleavage or translational repression, whereas trans‐acting small interfering RNAs (ta‐siRNAs) are secondary small RNAs originating from non‐coding TAS gene transcripts, whose production is triggered and guided by specific miRNAs through a tightly regulated pathway (Zhan and Meyers 2023). In trichome development, miR156 plays a key role by targeting and repressing SQUAMOSA PROMOTER BINDING PROTEIN‐LIKE (SPL) genes, which are negative regulators of trichome formation in *Arabidopsis*. As plants mature, their miR156 levels decrease, while SPL levels steadily rise, correlating with fewer trichomes on stems and flowers (Yu et al. 2010). Trichome density changes in response to overexpression of miR156 or miR156‐resistant SPLs. Additionally, miR171 regulates LOST MERISTEMS (LOM) genes, which interact with SPLs; decreased LOM levels reduce trichome density, while resistant LOM expression enhances it (Xue et al. 2014). miR319a also influences trichome density by targeting TCP transcription factors; overexpression of miR319a increases trichome density, whereas repression via STTM (short tandem target mimic) reduces it (Fan et al. 2020). In cotton, miR828 targets MYB family genes, such as *GhMYB2* and *GhMYB3*, similar to GL1 in *Arabidopsis*, a key player in trichome formation (Wan et al. 2016). Targeted cleavage of the respective mRNAs by miR828 lowers their levels and initiates the production of another group of small RNAs, called ta‐siRNAs, through a gene like TAS4. These ta‐siRNAs then cooperate with proteins called ARGONAUTEs to form complexes (RISCs) that can further block or destroy other related mRNAs (Mi et al. 2008). Apart from small RNAs, other post‐transcriptional mechanisms, such as alternative splicing, modulation of mRNA stability and mRNA transport, have been linked to trichome development. For instance, in *Brassica rapa*, the BrAN gene produces two different splice variants, lhd1 and lhd2, where lhd1 consists of a second intron and promotes the expression of the GL3 gene in *Arabidopsis* (Xin et al. 2022; Friede et al. 2017).

## 3 Defence Mechanism of Trichome

### 3.1 Trichomes Against Biotic Invaders

Non‐glandular trichomes may seem small and unassuming, but they serve as a physical defence, providing a protective shield for the plant and making it more difficult for herbivorous insects to move or feed (Karabourniotis et al. 2020). Especially when young, they can even mimic the look of glandular trichomes, though they do not secrete terpenoids. Still, they often store phenolics early on, adding another layer of subtle protection. Their density, length and stiffness play a crucial role in creating an unfriendly environment for these pests, often reducing their ability to access the epidermis and lay eggs (Levin 1973) (Figure 3). Studies in *Arabidopsis* have shown that plants with fewer trichomes are more vulnerable to insect attacks, such as those from *Spodoptera littoralis*, emphasising the structural role of trichomes in defence (Hauser 2014) (Figure 3). On the other hand, herbivores and microbial pathogens can be deterred, intoxicated or even killed by the wide range of chemical defence strategies, including secondary metabolites secreted by glandular trichomes, such as terpenoids, acyl sugars and flavonoids. In *Byblis guehoi*, two types of glandular trichomes, stalked and sessile, work together to help the plant catch and digest prey. The stalked trichomes secrete a sticky mucilage that traps insects, while the sessile trichomes release digestive enzymes and absorb nutrients through endocytosis (Poppinga et al. 2022). The sessile trichomes have specialised cuticle pores that enable them to absorb large molecules, such as proteins. Once absorbed, the nutrients are carefully transported to specific cells in the epidermis and mesophyll. This division of labour optimises prey digestion and nutrient absorption, demonstrating advanced carnivorous adaptations (Płachno 2023). Tomato (*S. lycopersicum*) type VI glandular trichomes produce volatile terpenes, including methyl ketones and α‐pinene, which defend against whiteflies and aphids (Bleeker et al. 2009). Similarly, acyl sugars secreted by trichomes in wild tomatoes (*S. pennellii*) exhibit insecticidal and anti‐ovipositional effects against multiple insect species (Vendemiatti et al. 2024). The regulatory network involving *wo* and *SlMYC1* in tomato trichome development plays a pivotal role in enhancing pest resistance by ensuring the production of glandular trichomes that produce insect‐repellent chemicals (Shi et al. 2025). Another report on tobacco (*Nicotiana benthamiana*) depicts that the accumulation of O‐acyl sugars in the trichomes enhances resistance against whiteflies. Overexpression of acyl sugar biosynthesis genes in *N. tabacum* enhances its resistance against *Bemisia tabaci* (J. X. Wang, Han, et al. 2025). Tea is a common beverage whose production is greatly hampered by grey blight (Muraleedharan and Chen 1997). Although the Thaumatin‐like protein (TPL) and kruniz type protease inhibitor (KPI) trigger the jasmonic acid or salicylic acid pathway for resistance against pathogen infection (P. Li et al. 2022). Trichomes can also contribute to indirect defences by attracting the natural enemies of herbivores. In wild cotton (*Gossypium hirsutum*), glandular trichomes release volatile scents that attract beneficial insects like *Orius insidiosus*, which prey on common pests such as thrips and aphids (Kessler and Baldwin 2001) (Figure 3). This tritrophic interaction enhances plant resistance by integrating physical and chemical defence traits with ecological dynamics. According to a study, the trichome shape, plant growth stage and insect behaviour all affect the trichome‐based defence in *Phaseolus vulgaris*. Using live capture assays, it can be concluded that the highest insect mortality was observed during the leaf stage, especially on the lower leaf surface with dense trichomes (Figure 3). Real‐time monitoring and microscopy revealed that insects were mainly trapped during landing and feeding, with their mouthparts and ovipositors being the most affected, hinder feeding and reproduction and often leading to death or reduced fitness (Xing et al. 2017). In *Cucumis sativus*, one trichome development‐related gene, *CsTM*, a lectin receptor‐like kinase, interacts with *CsTIP1* and helps achieve resistance against aphid pests by increasing cytoplasmic H_2_O_2_ levels (S. Yang, Xue, et al. 2025). In addition to insect herbivory, trichomes play a vital role in resisting bacterial and fungal pathogens. Trichome secretions often possess antimicrobial properties that inhibit the growth of pathogens such as *Pseudomonas syringae* and *Botrytis cinerea*. Studies have shown that *Arabidopsis* trichomes produce ROS in response to pathogen attack, contributing to localised defence responses. Some trichomes release or store powerful natural chemicals, like phenolics and flavonoids, that can kill or inhibit bacteria, fungi and herbivores (Benikhlef et al. 2013).

**Figure 3:**
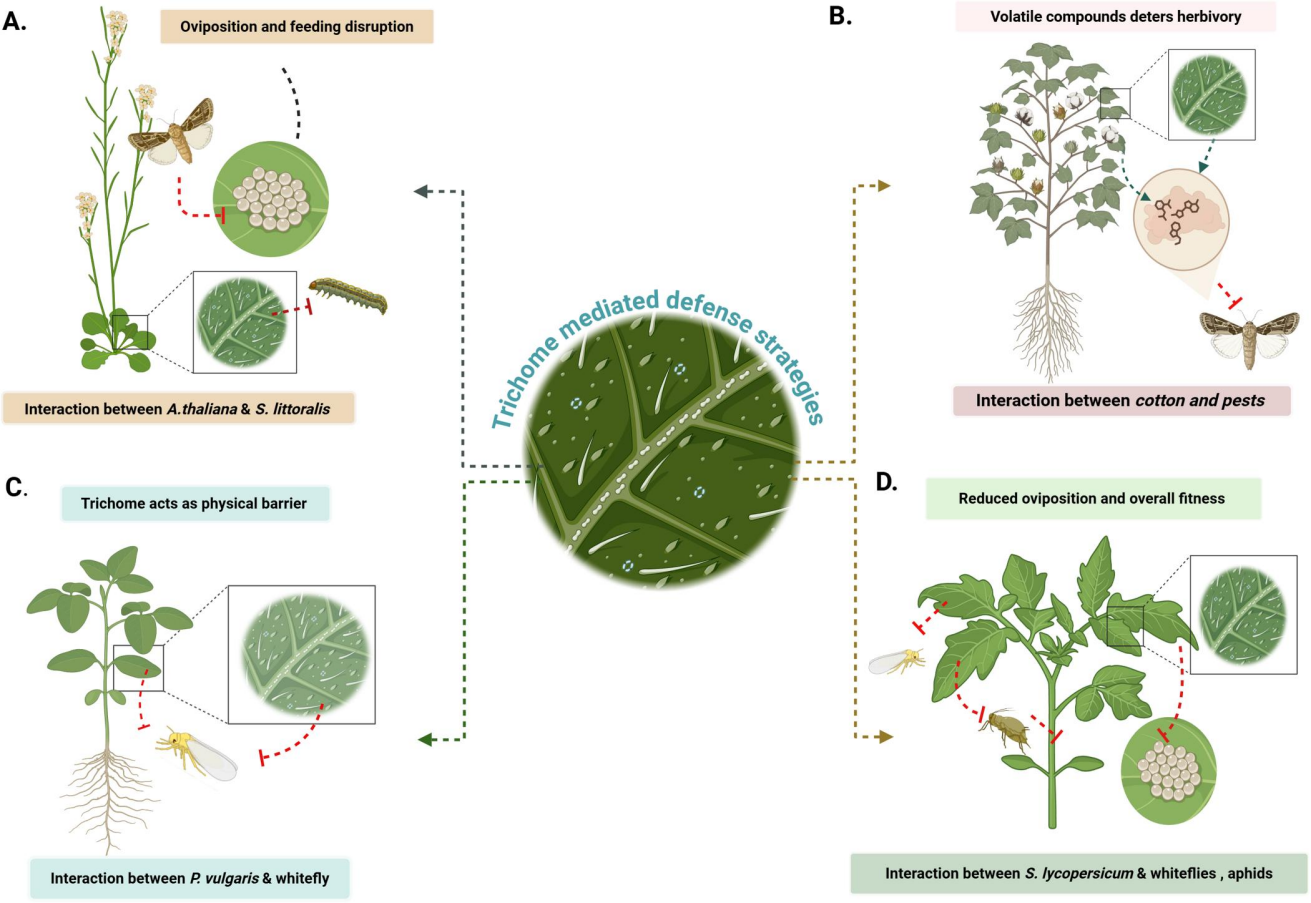
Trichome‐mediated defence strategies against insect pests through physical and chemical mechanisms. Pictorial representation of trichome‐mediated defence strategies in plants against insect herbivores and pests. (A) Trichomes interfere with insect oviposition and feeding behaviour, reducing egg deposition and larval establishment, as illustrated in the interaction between *Arabidopsis thaliana* and *Spodoptera littoralis*. (B) Glandular trichomes synthesise and release volatile organic compounds (VOCs) and other secondary metabolites that deter herbivory and influence pest behaviour, exemplified by cotton–insect interactions. (C) Non‐glandular trichomes act as a physical barrier, restricting insect movement, feeding and host colonisation, as shown in the interaction between *Phaseolus vulgaris* and whiteflies. (D) Increased trichome density and associated defensive metabolites reduce oviposition, feeding damage and overall pest fitness in *Solanum lycopersicum* (tomato), thereby enhancing plant resistance against whiteflies and aphids. Together, these mechanisms highlight the multifaceted role of trichomes in plant defence against a wide range of insect pests. Created in Biorender.com. [Color figure can be viewed at wileyonlinelibrary.com]

### 3.2 Trichomes as Frontline Defenders Against Abiotic Challenges

Plants face biotic stresses and must contend with numerous non‐living (abiotic) stresses in their environment. Dense trichome layers, which serve as the physical defence system, act as natural shields for plants, enhancing their ability to withstand harsh conditions, including drought, intense sunlight and elevated temperatures. According to S. Li et al. (2018), plants possessing dense glandular trichomes exhibited a wide range of chemical defence, resulting in less ozone‐induced damage, stronger photosynthetic performance and the release of fewer LOX‐derived stress volatiles, such as hexenals and hexenols. Their ozone fumigation studies and chemical analyses indicated that these trichomes function as a ‘biochemical sunscreen’, actively detoxifying ozone at the leaf surface and providing localised protection (S. Li et al. 2018). As ozone levels continue to rise due to pollution, this defence mechanism may become even more crucial in helping plants survive hostile environments. Recently, a comparative study between the two congeneric species of *Croton*, consisting of trichome‐dense (*Croton blanchetianus*) and less trichome (*Croton adenocalyx*), showed that the former possesses higher water retention capacity due to the presence of stellate trichomes and sclereids along with improved leaf gas exchange capability, which makes the plant resistant against drought stress (Araújo et al. 2024). In tobacco (*N. tabacum*), through gene expression analysis, microscopy and metal accumulation assays, it was found that *NtJAZ10* promotes glandular trichomes, which upregulate stress‐responsive genes against cadmium (Cd) stress, including PR proteins, glutathione‐related enzymes and metal‐binding proteins and by increasing ABA level in the plant (Harada et al. 2010; S. Wang, Cui, et al. 2025). Trichomes also excrete cadmium, reducing its toxicity in leaf tissues, highlighting their key role as localised detoxification centres that protect against abiotic stress and strengthen the plant's defence against herbivores (H. Zhang et al. 2021). Tea trichomes provide an excellent means of defence against excessive water loss, UV exposure and extreme sunlight. In a trichome‐rich tea variety, a trichome‐associated gene, *CsTBR*, helps mitigate harsh light exposure through trichome‐mediated light reflection (P. Li et al. 2022). Additional research on *Conyza blinii* established a correlation between increased trichome density and UV rays. An appropriate intensity of UV rays upregulates *CbMYB108*, which in turn increases capitate glandular trichome formation by targeting CbTTG1 and diterpene biosynthesis via upregulating genes associated with diterpene biosynthesis pathways (Zhan et al. 2024). Studies have shown that *C. sativa* grown in hotter and drier environments develops high‐density trichomes to enhance secondary metabolite production, resulting from a metabolic shift under drought conditions (Tanney et al. 2021; Sharma et al. 2025). *Colquhounia vestita*, a Chinese‐Himalayan shrub that grows at high altitudes and is constantly exposed to harsh sunlight and low temperatures, produces specialised chemical compounds for survival. Using laser microdissection and UPLC–MS/MS, researchers isolated and analysed trichomes from *C. vestita* and identified three major diterpenoid acids, most notably polyalthic acid, that enhance plant tolerance to UV radiation and cold stress. Experiments in *A. thaliana* demonstrated that polyalthic acid treatment enhanced seedling survival, mitigated oxidative damage, preserved photosynthetic efficiency and induced stress‐responsive gene expression (Tang et al. 2020). Moreover, Karabourniotis et al. (1993) demonstrate that leaf hairs (trichomes) on *Olea europaea* (olive) leaves defend against the harm caused by UV‐B rays. Through experiments comparing normal and de‐haired leaves exposed to moderate UV‐B radiation, researchers found that de‐haired leaves exhibited a significant and irreversible reduction in photosystem II efficiency, along with increased signs of damage, including epidermal browning and altered chlorophyll fluorescence, indicating disruptions in photosynthesis. In contrast, normal leaves with intact trichomes remained unaffected (Karabourniotis et al. 1993). In a study on *Schizonepeta tenuifolia*, salt stress (0–100 mM) reduced growth, whereas antioxidant assays (DPPH, ABTS) showed an increase in phenolic content at 25 mM. Salt stress led to a rise in glandular trichome density and a shift in exudate composition (GC–MS), with reduced pulegone and elevated stress‐induced esters (Y. Zhou et al. 2018). These experiments demonstrate how trichomes adapt structurally and chemically to protect plants against various forms of abiotic stress. Researchers found that *Dendrobium* species with leaf trichomes absorb more water through foliar uptake but lose it faster than species without trichomes. Experiments measuring water content, storage, retention time and surface wettability showed that trichomes improve water absorption but reduce retention, highlighting water‐use strategies among epiphytic orchids (Z.‐L. Pan, Guo, et al. 2021). Surprisingly, heat stress at 37°C in tomato results in a shift from non‐glandular type V trichomes to glandular type IV trichomes, which increases the production of acyl sugars, helping to achieve resistance to herbivores (Säbel et al. 2023). This type of crosstalk between abiotic and biotic stress can be of utmost importance for biotechnology in producing highly demanding stress‐resilient crops.

### 3.3 Secretory Activity and Metabolite Production of Trichomes

Trichome‐derived secondary metabolites act as chemical barriers against herbivores, insects and pathogenic microorganisms. It also possesses antimicrobial properties that prevent the growth of bacteria, fungi and viruses on the plant surface. Thus, trichome‐associated secondary metabolites constitute an important component of the plant's integrated defence system (Schilmiller et al. 2008). Trichome secretory cells function like chemical factories, utilising organelles such as the endoplasmic reticulum, plastids and mitochondria to produce a diverse range of metabolites (Tanney et al. 2021). The synthesised products are transported through plasmodesmata or apoplast in case of cell‐to‐cell transport, and also via ATP‐binding cassette transporter (especially MDR type) or H+ antiporter (gradient‐dependent secondary transport) into subcuticular cavities, formed by detaining a thick cuticle from the cell wall, creating a temporary storage pocket (Yazaki 2005; Xiao et al. 2025). In many plants, this pocket eventually opens or bursts to release the stored substances, primarily when pressure builds up from accumulating secretions (Han et al. 2022). The process is tightly controlled by a complex network of transcription factors, hormones and environmental factors, such as light and water, that influence trichome density, maturity and activity, thereby affecting metabolite production. The major classes of metabolites include terpenes, fatty acid derivatives, alkaloids, phenylpropanoids, polyketides and mixed‐type compounds, among others (Figure 4). Terpenoids are the most common and diverse group of compounds secreted by glandular trichomes, including monoterpenes, sesquiterpenes and diterpenes like artemisinin in *Artemisia*, cannabinoids in *Cannabis* and terpenes in tomato and peppermint (Contreras‐Avilés et al. 2024). Other beneficial compounds, such as phenylpropanoids and polyketides found in some types of basil, are produced by special glandular structures called peltate glands, which synthesise both terpenes and phenylpropanoids, including chavicol, eugenol, methylchavicol and methyleugenol. These are formed from simple alcohol‐based molecules through a series of chemical transformations, including acetylation, reduction and methylation (Gang et al. 2002). Hops (used in brewing beer) are another good example. Their trichomes produce a key compound, xanthohumol, a prenylated polyketide. They also produce smaller amounts of ‘bitter acids’ such as humulone, which contribute to beer's flavour. Researchers have even identified the specific enzyme (a methyltransferase) that helps make xanthohumol (Clark et al. 2013). Polyphenols, such as flavonoids (kaempferol, quercetin) and tannins, act as antioxidants and antimicrobial agents, offering protection to plants, including tomatoes, members of the Moraceae family. Acyl sugars, produced in trichomes by esterifying sugars or sugar alcohols with fatty acids, form sticky coatings on plants like tobacco and tomato, where they deter pests (aphids, whiteflies) by trapping, immobilising or poisoning them, thus playing a key role in plant defence (Nandi et al. 2022). Glandular trichomes also produce defensive proteins, such as chitinases and hydrolases, to protect against pathogens and pests (Vaghela et al. 2022). In *Croton gratissimus*, lepidote, glandular trichomes and laticifers actively produce and store compounds such as alkaloids, phenolics and lipids, forming a natural chemical defence system (Naidoo et al. 2023). Nitrogen‐containing compounds with highly bioactive properties, such as nicotine in tobacco and newly reported alkaloids in several Moraceae species, serve as potent deterrents and toxins for herbivores (Zador and Jones 1986). Very recently, a gene Tq HD1 from thyme (*Thymus quinquecostatus*) was overexpressed in tomato, which not only increased the glandular trichome density but also elevated the bioactive terpenoid profile (Y. Zhang et al. 2025).

**Figure 4:**
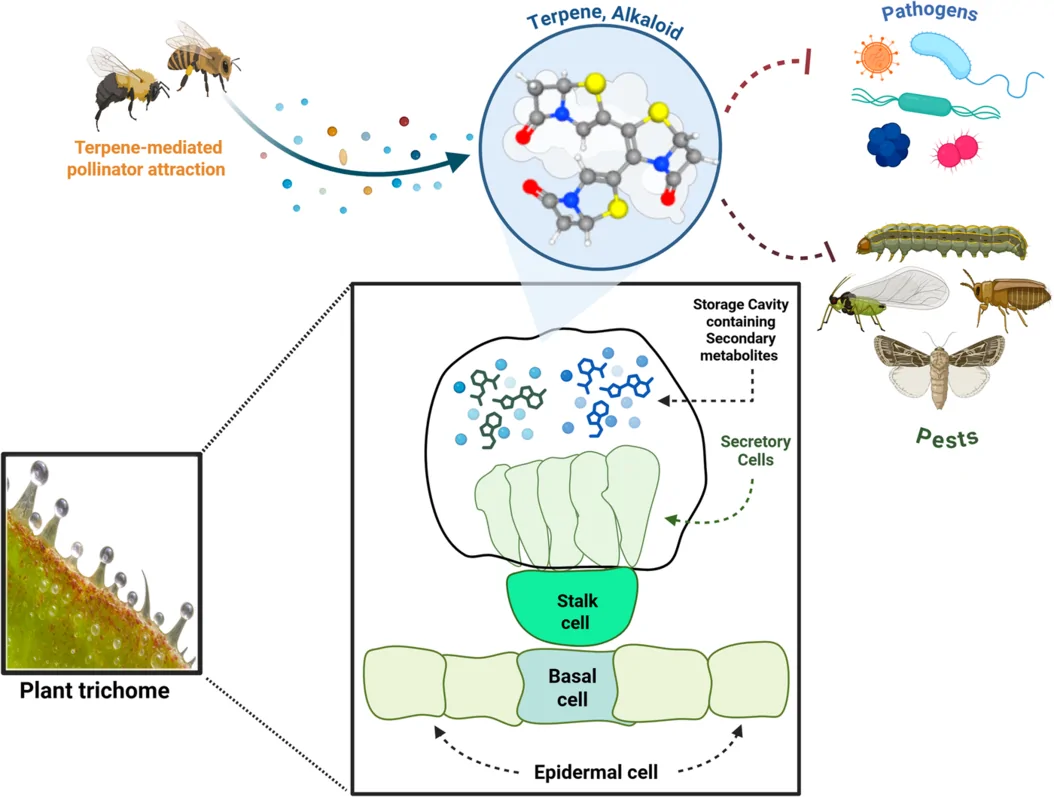
Trichome‐derived secondary metabolites in plant defence and ecological interactions. Plant trichomes produce and store secondary metabolites, which include terpenes, alkaloids, phenylpropanoids, polyketides and fatty acid derivatives. This diverse array of secondary metabolites provides chemical defence against pathogens, develops physical barriers and mechanical strength via lignin synthesis, strengthens antimicrobial properties, attracts pollinators and maintains ecological signalling (Jin et al. 2025). Created in Biorender.com. [Color figure can be viewed at wileyonlinelibrary.com]

## 4 Research Gap, Emerging Technological Advances and Upcoming Avenues for Trichome Research

Plant trichomes are critical guardians of the green world, performing diverse roles contributing to plant survival, adaptation and ecological success. Development of trichome is tightly controlled by environmental stimuli such as temperature, light intensity, atmospheric CO_2_ and water availability. High temperature increases trichome density to protect against heat stress, whereas low‐temperature‐driven cold damage is mitigated by increased trichome density (Bittencourt‐Oliveira et al. 2012). Heat stress and excessive UV ray exposure can be reduced by enhancing trichome formation, which helps reflect excess radiation and lower leaf temperature. Elevated CO_2_ showed species‐specific responses in trichome density (Karowe and Grubb 2011). Overall, trichomes help as a multifunctional adaptive structure that improves plant survival under diverse environmental stresses.

This review examines three key aspects of trichomes in various plants: development, defensive functions and secretory activities. However, some areas still require further understanding (Box 1). The production of plant‐based, valuable synthetic compounds using a heterologous system hinders their commercialisation due to high costs and low yields. The use of plant systems, such as tomato and tobacco, for the synthesis of therapeutic compounds is also not widely accepted due to their slow growth and genetic complexity (Gomes et al. 2016). Future studies should focus on single‐cell analyses and spatial multi‐omics approaches, such as transcriptomics, proteomics, metabolomics and epigenomics, not only to unravel gene expression dynamics and developmental trajectories between glandular and non‐glandular trichomes but also to allow trichome reprogramming, thereby making the plant system a sustainable production hub (H. Chen et al. 2024; P. Zhou et al. 2021; S. Sun et al. 2023; Fesenko and Edwards 2014). For example, single‐cell RNA sequencing has revealed that the differential expression of specific transcription factors, such as HOX3 and MYB25‐like, governs fibre differentiation in cotton (Y. Qin et al. 2022). Another time‐dependent transcriptome data set suggests that HOX3 is associated with non‐glandular trichome elongation, while bHLH1 is involved in glandular trichome formation in *C. sativus* (M. Dong et al. 2022). Comparative transcriptome analysis involving hairless (CYQ) and hairy (BDM) varieties of tea plant revealed that MYB 75 and NOK were upregulated in the hairy group and help in trichome initiation, whereas ATML1, SPL6 and SPL12 were upregulated in the hairless group, suggesting their possible role in trichome suppression (L. Chen et al. 2022). By combining this information with studies on chromatin structure and epigenetic changes, we can gain a deeper understanding of how non‐coding RNAs, histone modifications and DNA methylation regulate trichome growth and their ability to adapt to various functions. Studying how phytohormones, such as gibberellins, cytokinins, auxin, jasmonic acid and brassinosteroids, interact at the molecular level enhances trichome engineering. As we learn more about these interactions, targeting specific pathways for enhancing plant defence and metabolite production will become easier. Genome editing tools like CRISPR–Cas will speed up identifying and using beneficial genetic traits that control trichome characteristics and their associated biosynthetic pathways (Muto and Matsumoto 2022; K. Chen et al. 2019). A study on hybrid poplar showed that CRISPR‐mediated multiplex editing targets MYB186 and MYB138, resulting in a trichomeless phenotype with abolished triterpene synthesis, which further provides some hints on the relationship between poplar trichome and triterpene synthesis (Bewg et al. 2022). In another study, CRISPR‐mediated knockout of the H3 and H4 genes in tomato revealed their roles in trichome development, with the h3/h4 double mutant showing a decreased number of type I trichomes (Kim et al. 2025). Nevertheless, CRISPR/Cas systems now enable precise, causal tuning of plant trichomes, both their abundance and secretory function, by directly editing the regulators that initiate or pattern these epidermal outgrowths. This delivers a practical route from gene discovery to deployable trait engineering in crop surfaces and specialised metabolite factories (Figure 5).

Box 1 **Outstanding questions for future research.**

- How do evolutionary forces shape the trichome patterning across different plant lineages?
- In what ways does the crosstalk between environmental stressors and epigenetic modifications contribute to the development of stress resistance in crop plants via trichome‐related traits?
- By what mechanisms do plants determine the appropriate type of trichome to deploy in response to diverse and overlapping abiotic and biotic stress conditions?
- How do secondary metabolites derived from plant trichomes contribute to the advancement of synthetic biology and metabolic engineering applications?
- How can the adaptive plasticity of trichome development be harnessed to engineer climate‐resilient crop varieties?
- What insights do single‐cell omics and CRISPR‐Cas technologies provide for the targeted enhancement of trichome‐derived traits, thereby improving plant survivability under stress conditions?

**Figure 5:**
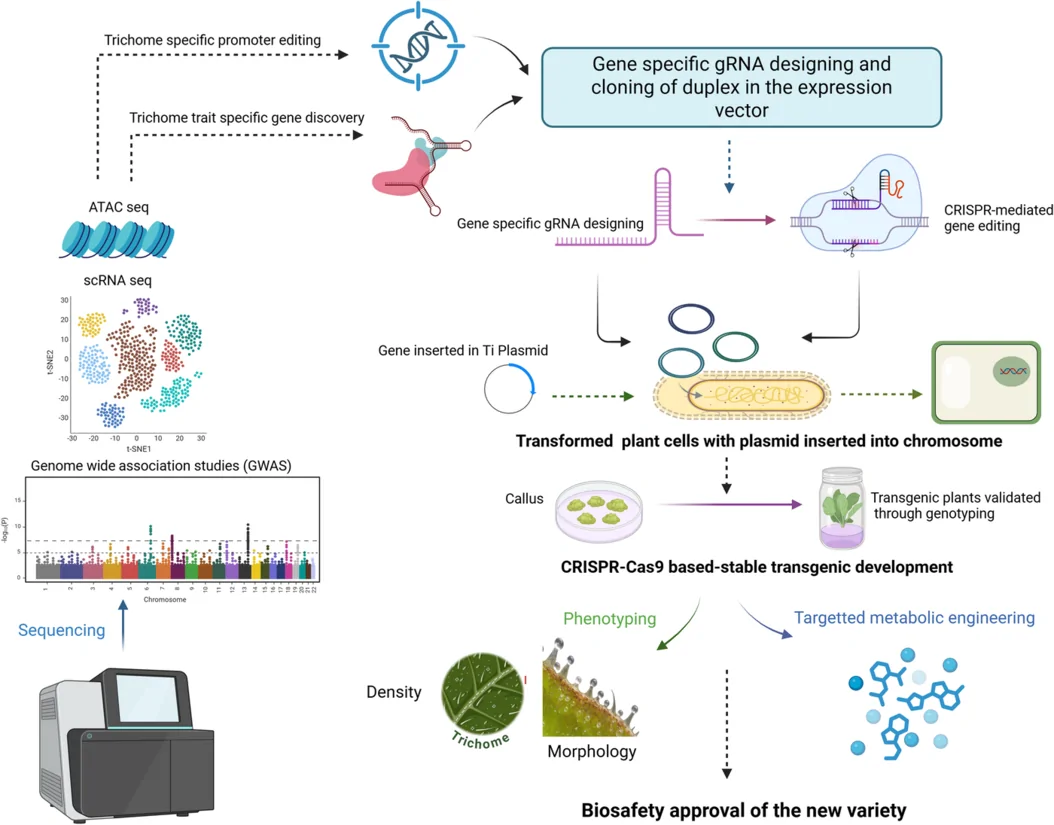
Pipeline for trichome gene discovery, CRISPR–Cas9‐mediated editing and improved trait development. Schematic overview linking gene discovery to CRISPR–Cas9 editing and deployment in trichome engineering. Candidate trichome genes and promoters are identified from multi‐omics data sets—ATAC‐seq (chromatin accessibility), single‐cell RNA‐seq (cell‐type expression) and GWAS of sequenced accessions—followed by gene‐specific guide RNA (gRNA) design and cloning a gRNA duplex into an expression vector. The construct is assembled in a Ti plasmid and delivered to plant cells (*Agrobacterium*‐mediated transformation), after which the transgene is inserted into the host chromosome. Edited tissues form callus and are regenerated into plants; putative transformants are validated by genotyping. Stable CRISPR–Cas9 lines are advanced for phenotyping of trichome density and morphology, as well as for targeted metabolic engineering, which requires biosafety approval of the new variety. All icons are illustrative and not to scale. ATAC‐seq, assay for transposase‐accessible chromatin sequencing; GWAS, genome‐wide association study; gRNA, guide RNA; scRNA‐seq, single‐cell RNA sequencing; Ti, tumour‐inducing (*Agrobacterium*) plasmid. Created in Biorender.com. [Color figure can be viewed at wileyonlinelibrary.com]

Expanding molecular research into non‐model and crop species, such as cucumber, tomato, cotton and tea, will help reveal conserved and unique regulatory systems that control trichome development and function. By examining how species‐specific pathways, gene duplications and evolutionary changes contribute to trichome diversity, we can gain a deeper understanding of trichome evolution across different plant lineages. Another exciting possibility is using trichomes as natural ‘biofactories’ (Schilmiller et al. 2008). As the human population increases, a sharp contrast between demand and supply becomes evident, which can be mitigated through plant metabolic engineering that utilises established biosynthetic pathways (Selma et al. 2023). By engineering the metabolic processes of glandular trichomes using gene regulatory elements, enzyme modifications and transporters, we can significantly enhance the production of valuable products, including pharmaceuticals, nutraceuticals, pesticides and flavour/aroma compounds. Metabolic engineering of the type VI glandular trichome, including the TcCDS, Tc ADH2 and TcALDH1 genes in tomato, facilitates the production of an insecticide (pyrethrins) that is less toxic to mammals. Its short‐term persistence makes it eco‐friendly, providing strong insecticidal resistance (Y. Wang, Wen, et al. 2022). Studying the variation in trichomes across different plant species, their genetic causes and their role in adapting to the environment will provide a deeper understanding of how plants interact with their surroundings and develop resilience (J. Pan, Zhang, et al. 2021). Studies on the adaptive evolution of trichomes further strengthen their importance in agriculture. Increased density and height of the non‐glandular trichomes provide tolerance to cold stress while increasing the intake of water vapour, reducing the penetration of solar radiation, and helping the plant survive severe drought and heat damage. Moreover, high‐density glandular trichomes with increased length pave the way for insect‐pest‐resistant breeding (Y. Zhang et al. 2020). Thus, the evolution of trichome structure, chemical properties and ecological role provides valuable insight into plant survival strategies.

## 5 Conclusion

This review has synthesised current knowledge surrounding three principal domains of trichome biology: their development, defensive mechanisms and secretory capacities across diverse plant taxa. Understanding how gene duplications, neofunctionalization and selective pressures have shaped trichome diversity across evolutionary time offers a promising framework for comparative developmental biology. From an ecological and evolutionary standpoint, studying the morphological and chemical diversification of trichomes, their underlying genetic drivers and their roles in plant–environment interactions will enrich our understanding of plant adaptive strategies and resilience mechanisms. These insights hold profound implications for fundamental plant science and applied sectors such as crop improvement, integrated pest management and sustainable agriculture. Nevertheless, plant trichomes are more than simple surface structures; they are dynamic and multifunctional interfaces between plants and their environments. Leveraging their full potential will require an integrative, multi‐disciplinary approach, linking cutting‐edge technologies with ecological and evolutionary perspectives.

## Funding

The authors have nothing to report.

## Conflicts of Interest

The authors declare no conflicts of interest.

## Data Availability

Data sharing is not applicable to this article as no data sets were generated or analysed during the current study.
